# Mental health consequences of contemporary cannabis use in Europe: potency, patterns of use, and health system context

**DOI:** 10.3389/fpsyt.2026.1778831

**Published:** 2026-06-10

**Authors:** Justyna Śniadach, Sylwia Szymkowiak, Przemysław Osip, Wiktor Orlof, Napoleon Waszkiewicz

**Affiliations:** 1 Department of Psychiatry, The Faculty of Medicine, Medical University of Białystok, Białystok, Poland; 2 Hipolit Cegielski Medical Centre (Centrum Medyczne HCP), Poznań, Poland

**Keywords:** cannabis, cannabis use disorder, Europe, health system readiness, psychiatric disorders, psychosis, regional disparities, THC potency

## Abstract

**Background:**

Cannabis use has increased across Europe over the last decade, accompanied by growing availability of high-potency THC products and rising psychiatric presentations. Despite these trends, European countries differ substantially in monitoring systems, clinical preparedness and public health responses. These disparities are especially visible between Western Europe, which has more developed pharmacovigilance systems, diagnostic networks, and addiction psychiatry services, and other regions. Compared with other regions, in Central and Eastern Europe cannabis-related psychiatric complications remain systematically underestimated.

**Methods:**

We conducted a narrative review of peer-reviewed literature, epidemiological reports and national health data published between 2010 and 2025. Sources included EMCDDA, WHO Europe, national statistical agencies and clinical studies on psychiatric outcomes associated with cannabis use.

**Findings:**

Cannabis is the most commonly used illicit psychoactive substance in Europe, with higher prevalence in Western countries but rapid growth across Central and Eastern Europe. The proliferation of high-potency cannabis (>15–25%) is consistently linked to elevated risks of psychosis, anxiety, panic symptoms, derealisation episodes and cannabis use disorder. Western Europe demonstrates better detection of cannabis-related psychiatric presentations, whereas Central and Eastern Europe show a hidden clinical burden driven by stigma, limited screening, inconsistent diagnostic practices and the absence of structured monitoring systems. Health systems vary widely in preparedness, particularly regarding early identification of high-risk use, addiction psychiatry integration and routine collection of psychiatric outcome data.

**Interpretation:**

Cannabis use is linked to a growing mental health burden in Europe. Stronger THC products and uneven health systems make this problem worse. In Central and Eastern Europe, many cannabis-related mental health problems are missed or not reported. Better monitoring, clearer diagnostic rules, and coordinated action across Europe are needed.

## 1 Introduction

Cannabis use remains the most prevalent form of illicit psychoactive substance consumption in Europe and continues to increase. In 2024, an estimated 22–24 million adults in Europe reported cannabis use, corresponding to approximately 8% of the adult population. These estimates refer to use within the past 12 months, representing an almost 40–50% increase over the past decade, compared with approximately 15–17 million users (5–6%) around 2014 (1–3). Where available, data on past-month use show similar upward trends, although such estimates vary depending on the population and methodology. The upward trajectory is evident both in the general population and among young adults, a group in which availability, normalization, and perceived low risk of cannabis use have been steadily increasing for more than a decade (4, 5). Importantly, daily or near-daily cannabis use represents a key risk factor for adverse outcomes, including dependence and psychiatric complications (4).

In parallel, the European cannabis market has undergone a profound shift toward high-potency products. Whereas herbal cannabis in the early 2010s typically contained single-digit THC (Δ9-tetrahydrocannabinol) concentrations (6–10%), contemporary markets in many European countries are now dominated by products exceeding 20–25% THC, with some preparations reaching even higher levels (6, 7). Depending on the source and country i5 estimates often range between ~15% and 25%. This doubling to tripling of THC dependence severity, represents not merely a quantitative change but a qualitative transformation of exposure, with direct implications for psychiatric risk, dependence severity and service demand.

For the purpose of this review, European regions were categorized as follows:

Western Europe (United Kingdom, Netherlands, France, Belgium, Germany),

Northern Europe (Denmark, Finland, Sweden, Norway),

Southern Europe (Spain, Italy, Portugal, Greece, Cyprus, Malta),

Central Europe (Poland, Hungary, Czech Republic, Slovakia),

and Eastern Europe (Romania, Bulgaria, Lithuania).

This classification was based on European Monitoring Centre for Drugs and Drug Addiction (EMCDDA) (2, 7).

This review examines how rising THC potency reshapes the psychiatric burden in Europe, with a particular focus on health system readiness and regional disparities in detection, reporting.

These developments have been accompanied by a growing psychiatric burden. Clinical and population-based studies report increasing numbers of anxiety episodes, affective disorders, and cases of first-episode psychosis associated with cannabis use (8, 9). The highest risk is observed among young individuals who initiate use before full neurobiological maturation, as well as among those with genetic and environmental vulnerability factors (10–12).

Cannabis use is not the same across Europe, while data quality also differs between regions, for example in Western Europe, monitoring systems are better developed. Countries such as Germany, the Netherlands, Portugal, and France collect more reliable data on cannabis use and mental health outcomes (3, 5, 13). Because of this, cannabis-related psychiatric problems are more often identified. In Central and Eastern Europe, the situation is worse, as countries such as Poland, Romania, Bulgaria, and Hungary report lower prevalence of cannabis use, although these estimates are likely underestimated due to stigma, more restrictive drug policies, limited epidemiological surveillance, and reduced access to specialist psychiatric care (4, 14, 15). This has clinical consequences as cannabis use disorder is often recognised late, psychotic symptoms may be classified as primary disorders. Treatment is delayed or not offered.

Over the past two decades, cannabis products have become much stronger. Meta-analyses show that average THC levels have increased more than twofold, while CBD (cannabidiol) levels have decreased (16). This matters, because CBD may have partly protective effects, although the evidence remains mixed and still debated. The balance between THC and CBD has clearly changed. Similar patterns are seen on regular markets and on darknet platforms, where extracts and concentrates with very high THC levels are common, often reaching 60–80% THC (17, 18). Products with high THC and low CBD are linked to higher psychiatric risk. Acute psychosis, severe anxiety, and rapid development of dependence are reported more often with these products (6, 19, 20).Evidence from Finland, based on post-mortem toxicological analyses, indicated a short-term increase in cannabis-related findings, including THC detection, during the early phase of the COVID-19 pandemic (21). This may reflect temporary changes in substance availability and patterns of use during the pandemic rather than sustained long-term trends. These market changes occur faster than monitoring systems can follow. This is especially visible in Central and Eastern Europe, where market surveillance is limited and regulation allows online sellers to bypass local controls, increasing exposure to high-risk products.

Cannabis use is linked to many psychiatric problems, especially when stronger cannabis products are used. Studies show a higher risk of first-episode psychosis, anxiety, derealisation, mood problems, and cognitive impairment (22–25). This risk is higher in young adults and in people who start using cannabis during adolescence, when the brain is still developing (26–29).

Cannabis is used for two main purposes in Europe: under medical supervision for specific therapeutic indications, and non-medically for recreational or self-directed use. Both forms of use may be associated with adverse effects depending on the product, dose, frequency of use, and individual vulnerability. In recent years, cannabis has also increasingly been used for self-medication, particularly in Central and Eastern Europe, often without professional supervision, which may delay recognition of warning signs and increase the risk of long-term psychiatric complications (30–32). In recent years, more people have started using cannabis for self-medication, especially in Central and Eastern Europe, often without medical supervision, as a result, early warning signs are missed and the risk of long-term psychiatric problems increases (14, 33, 34).

In some European countries, psychiatry and addiction treatment are poorly connected. This includes Poland, Romania, and Hungary. In these countries, the real burden of cannabis-related problems is high. Health systems often struggle to diagnose and treat these conditions (5, 35, 36). Standard tools for assessing cannabis use disorder are not always used, there are too few specialists, stigma is still common and oordination between services is weak. This delays responses to risks linked to high-potency cannabis products.

## 2 Materials and methods

### 2.1 Search strategy

This study was conducted as a narrative review based on a targeted search of the literature addressing cannabis use in Europe, changes in THC potency, psychiatric consequences of cannabis use, and the readiness of health care systems to detect and treat these conditions.

The literature search was performed in PubMed, Scopus, Web of Science, and Google Scholar. Additional sources were also included, for example reports from the EMCDDA and European Union Drugs Agency (EUDA), data from national monitoring systems, WHO documents, strategic publications, and grey literature, defined as institutional reports and public health policy documents.

The search strategy employed the following keywords and their combinations: “cannabis,” “marijuana,” “THC potency,” “high-potency cannabis,” “Δ9-THC,” “first-episode psychosis,” “psychosis,” “psychiatric symptoms,” “anxiety,” “panic attacks,” “derealisation,” “depersonalisation,” “withdrawal,” “cannabis use disorder,” “CUD,” “emergency department,” “Europe,” “EMCDDA,” “psychiatric outcomes,” “public health,” “ICD-10,” “ICD-11,” and “treatment of cannabis-related disorders”.

Search strategies were adapted to the characteristics of individual databases using Boolean operators (AND/OR). Examples of database-specific queries included:

-PubMed: (“cannabis” OR “marijuana” OR “high-potency cannabis”) AND (“THC potency” OR “Δ9-THC”) AND (“psychosis” OR “first-episode psychosis” OR “anxiety” OR “panic” OR “ED visits” OR “CUD”) AND (“Europe” OR “EMCDDA”).
-Scopus: TITLE-ABS-KEY (“cannabis” OR “marijuana”) AND (“THC potency”) AND (“psychosis” OR “psychiatric symptoms” OR “CUD”) AND (“Europe”).
-Web of Science: TS = (“cannabis” OR “high-potency cannabis”) AND TS = (“psychosis” OR “FEP” OR “CUD” OR “anxiety”) AND TS = (“Europe”).
-Google Scholar: combinations of search terms including “cannabis Europe,” “THC potency increase,” “cannabis mental health outcomes,” “FEP and cannabis,” “ED presentations cannabis,” and “CUD epidemiology.”

In addition, reference lists of key articles were manually screened (hand-searching of references) to identify further relevant publications.

Publications published between 2010 and 2025 were included to capture the substantial increase in THC potency observed over the past decade and the most recent evidence on psychiatric outcomes associated with cannabis use. The review also incorporated European multicentre studies (including EU-GEI), epidemiological reports from the EMCDDA, and data from national systems monitoring health-related harms.

### 2.2 Inclusion criteria

Publications were included if they:

-were published between 2010 and 2025;
-focused on Europe or included comparative data involving European countries;
-provided information on THC potency, patterns of cannabis use, or mental health outcomes;
-described clinical consequences, including psychosis, anxiety symptoms, emergency department presentations, cannabis use disorder, withdrawal symptoms, mood disorders, or cognitive impairment;
-analysed diagnostic International Classification of Diseases, Tenth Revision (ICD-10), International Classification of Diseases, Eleventh Revision (ICD-11) or system-level aspects related to the treatment of individuals using cannabis;
-were peer-reviewed articles, large-scale reports (e.g., EMCDDA/WHO), or cohort studies.

### 2.3 Exclusion criteria

Publications were excluded if they:

-were non–peer-reviewed commentaries or opinion papers without empirical data;
-focused exclusively on social aspects without reference to mental health;
-were *in vitro* studies or purely pharmacological investigations;
-presented unclear methodology or insufficient clinical data;

originated outside Europe, unless they provided relevant comparative data on THC potency or psychiatric outcomes.

A summary of inclusion and exclusion criteria is presented in **Table 1**.

**Table 1:** Inclusion and exclusion criteria.

Inclusion criteria	Exclusion criteria
Publications from 2010–2025 addressing cannabis use and mental health outcomes.	Non–peer-reviewed publications, commentaries, and opinion papers without empirical data.
Studies and reports focusing on Europe or comparative analyses including European countries.	Publications focusing exclusively on social aspects without reference to mental health.
Data on THC potency, epidemiological trends, and patterns of cannabis use.	*In vitro* studies or purely pharmacological research without clinical relevance.
Studies describing clinical outcomes, including psychosis, first-episode psychosis, anxiety symptoms, derealisation, emergency department presentations, cannabis use disorder, withdrawal symptoms, mood disorders, and cognitive impairment.	Publications with unclear methodology, insufficient clinical data, or high risk of systematic bias.
Studies analysing diagnostic frameworks (ICD-10, ICD-11), treatment approaches, and system-level challenges in health care.	Studies conducted outside Europe, unless providing relevant comparative data on THC potency or psychiatric risk.
Peer-reviewed articles, large institutional reports (e.g., EMCDDA, WHO), cohort and epidemiological studies.	Publications not related to cannabis or its impact on mental health.

### 2.4 Article selection and bias minimisation

The initial search identified approximately 420 publications and reports. After screening titles and abstracts, 278 full-text articles were assessed for methodological quality and relevance. Ultimately, 92 publications with the highest clinical and epidemiological quality were included in the final review.

The selection process was conducted independently by two researchers from the authorship team with expertise in psychiatry and public health. Discrepancies were resolved through consultation with a third senior reviewer acting as an arbitrator.

**Figure 1:**
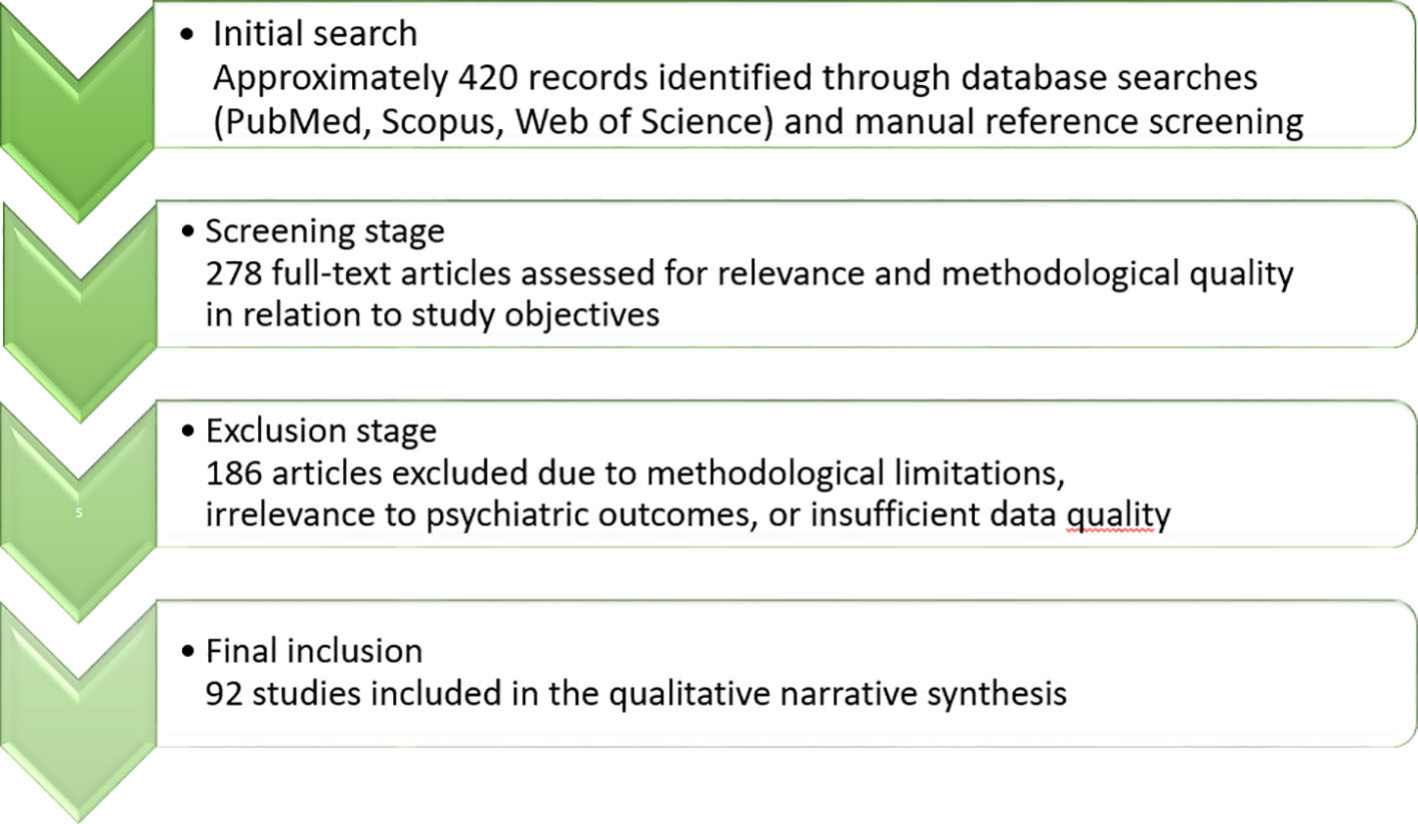
Flowchart of articles selection process.

Although a structured search strategy was applied, this study remains a narrative review. No formal risk-of-bias assessment or quantitative meta-analysis was performed. Conclusions are based on qualitative synthesis of the available evidence and should be interpreted in light of these limitations. The study selection process is illustrated in **Figure 1**.

Not all screened studies were individually cited in the manuscript; however, all contributed to the overall qualitative synthesis of evidence.; however, all contributed to the overall qualitative synthesis of evidence. A list of additional studies included in the final synthesis but not individually cited (n = 19) is provided in **Supplementary Material S1**.

## 3 Cannabis and mental health in Europe: results of the review

In the last 20 years, cannabis use in Europe has changed in several ways. Use has increased, but this is not the only change, as cannabis products have become stronger and user profiles have shifted. More people report mental health problems linked to cannabis use and health care systems are under increasing pressure. European reports point to clear regional differences, but also to shared trends across countries, including wider availability of high-potency products and growing social acceptance of cannabis, especially among young adults (2, 35). At the same time, the real clinical burden is likely higher than reported because. in many countries, especially in Central and Eastern Europe, cannabis-related mental health problems are often underdiagnosed.

This review shows that contemporary cannabis use in Europe is linked to a growing mental health burden that is often underestimated by health care systems. The analysis allowed the identification of three main axes determining the current clinical landscape:

1. Cannabis-associated psychiatric disorders in Europe, encompassing a spectrum ranging from acute anxiety reactions to psychotic disorders, affective disorders, cognitive disorders, and the development of Cannabis Use Disorder (CUD).
2. Increasing THC potency and the changing profile of cannabis products, constituting a risk factor for more severe clinical manifestations, increased toxicity, and greater burden on health care systems.
3. Marked regional differences in the recognition and recording of cannabis-related harms, particularly between Western Europe and Central and Eastern Europe.

In the following subsections, a detailed description is provided of the main categories of psychiatric disorders associated with cannabis use, their epidemiology in Europe, the role of increasing THC potency, and regional differences in the identification and treatment of clinical problems.

### 3.1 Mental disorders associated with cannabis use in Europe

Clinical and epidemiological evidence indicates that cannabis use is associated with a broad spectrum of mental disorders, ranging from acute anxiety reactions, panic attacks, and derealisation to persistent affective disorders, cognitive impairments, and the development of cannabis use disorder (23, 25, 37, 38). The risk is particularly elevated among young individuals, daily users, and those exposed to high-potency THC products (6, 11, 19). Neurobiological studies demonstrate that THC affects dopaminergic, glutamatergic, and endocannabinoid systems, thereby modulating vulnerability to psychotic disorders and affective disorders, including schizophrenia and related disorders, particularly in vulnerable individuals (27, 39, 40).

In Western European countries, well-developed early detection systems are in place, including screening in emergency departments, addiction treatment services, and pharmacovigilance frameworks (5, 41), which facilitate earlier identification of cannabis-induced psychotic disorders, acute anxiety or fear-related disorders, and cannabis withdrawal syndromes (42).

In Central and Eastern Europe, clinical detection remains limited due to stigma, insufficient professional training, and restricted addiction psychiatry infrastructure (14, 33). Fragmented registries, inadequate substance use screening, and a lack of specialised referral centres contribute to substantial underestimation of the true scale of the problem (15, 34). The scope and recognition of cannabis-associated mental disorders differ markedly across European regions Western and Northern Europe report the highest clinical burden, reflecting both greater availability of high-potency cannabis products and more developed case registration systems (9). By contrast, Central and Eastern Europe is likely characterised by pronounced underdiagnosis (14, 33, 34). Cultural normalisation, increased availability, and intensification of use patterns contribute to rising numbers of presentations in emergency departments and psychiatric outpatient services (41, 43), with similar trends observed in Southern European countries (15, 42).

In this context, it is important to note that the trends observed in Europe are also reflected in recent analyses of psychoactive substance use, including exposure to nicotine and substances commonly co-used with cannabis. Biomarker studies confirm that use patterns frequently involve polysubstance use, which further increases the risk of mental disorders and complicates the interpretation of epidemiological data (44, 45).

The most recent meta-analysis focusing on adolescent populations indicates that regular cannabis use is associated with an increased risk of anxiety symptoms, depressive symptoms, and psychotic symptoms, with the strongest effects observed among individuals who initiate use at a younger age (46). Although global in scope, these findings are consistent with European observations, where early initiation remains one of the most important risk factors for the severity of cannabis-associated mental disorders.

This comprehensive review integrating population-based, clinical, and neurobiological data confirms that cannabis use, particularly high-potency products and use initiated at a young age, is associated with an elevated risk of mental health problems, including anxiety or fear-related disorders, affective disorders, and overall deterioration in psychological functioning. These effects may result from both the direct impact of THC on neurotransmitter systems and interactions with stress exposure, genetic vulnerability, and environmental factors (47). Collectively, these findings reinforce the conclusion that cannabis is not a neutral substance with respect to mental health, particularly within European populations.

#### 3.1.1 Psychotic disorders and first-episode psychosis

The association between cannabis use and psychotic disorders is among the best documented relationships in psychiatric epidemiology. Numerous meta-analyses and population-based studies have demonstrated that regular cannabis use increases the risk of psychosis, particularly in the context of daily use and exposure to high-potency THC products (8, 10, 11).

The most recent meta-analysis focusing exclusively on adolescent populations confirms these findings, demonstrating a significantly elevated risk of psychosis among young cannabis users and highlighting the moderating role of factors such as frequency of use, THC potency, and familial liability (48).

The multicentre European Network of National Schizophrenia Networks Studying Gene–Environment Interactions study (EU-GEI) revealed marked regional differences, in cities such as London, Amsterdam, and Paris, where the market is dominated by products containing more than 15–20% THC, the risk of first-episode psychosis was nearly fivefold higher among daily users compared with non-users (8). The highest incidence rates of FEP were observed in large metropolitan areas with wide availability of high-THC cannabis, including Amsterdam, The Hague, London, south-east London, Paris, and Barcelona. Lower incidence rates were reported in cities with more limited access to high-potency products, for example Pamplona, Valencia, Bologna, Palermo, Rimini/Imola, and Sofia (9).

An inverse pattern was observed in several southern and Central and Eastern European cities, including Palermo, Madrid, Sofia, and Warsaw, where lower cannabis-attributable fractions likely reflected both historically lower product potency and restricted case detection systems (12).

Continued cannabis use following a psychotic episode is associated with an increased risk of relapse, longer hospitalisations, and poorer functional outcomes (49). Proposed neurobiological mechanisms include dysregulation of dopaminergic and glutamatergic systems, as well as altered endocannabinoid modulation, which together increase vulnerability to paranoid ideation, hallucinations, and thought disorder (28, 39).

In Norwegian psychiatric registries, cannabis-induced psychotic disorders are recorded as a stable and well-defined subtype of substance-induced psychotic disorders, allowing reliable monitoring of their population-level burden (50). These findings align with broader observations concerning neurobiological vulnerability reported in studies of psychoactive substance exposure conducted in Poland, including analyses of biomarkers related to various nicotine products. These studies similarly indicate substantial interindividual biological susceptibility to substance-related psychiatric outcomes (44).

#### 3.1.2 Anxiety or fear-related disorders, panic reactions, and derealisation

Acute anxiety reactions and panic attacks are among the most frequently reported adverse psychiatric effects associated with cannabis use. Users, particularly those who are inexperienced or exposed to high-potency THC products, may experience sudden panic attacks, derealisation, tachycardia, and intense fear, often resulting in emergency department presentations. Derealisation is considered a transient perceptual disturbance and should not be equated with a psychotic episode (19, 51, 52).

A recent meta-analysis focusing on adolescent populations demonstrated that cannabis use significantly increases the risk of anxiety symptoms and anxiety or fear-related disorders, with the strongest effects observed among individuals who initiated use at a younger age (53). The authors emphasise that adolescents constitute a particularly vulnerable group, likely due to neurobiological immaturity and heightened reactivity of stress-response systems. These findings are consistent with European observations, as countries ranging from the United Kingdom to the Netherlands report a rising number of acute anxiety and panic-related presentations among young users of high-potency products. This trend coincides with the expansion of THC concentrates and extracts across the European market (51, 53).

In Western Europe, including the United Kingdom, France, Belgium, and the Netherlands, a clear increase in cannabis-associated anxiety presentations has been documented, strongly correlating with the growing popularity of concentrates and high-potency products (41, 43). In Central and Eastern Europe, anxiety symptoms are also common; however, emergency department visits are far less frequently recorded. This pattern reflects underdiagnosis, insufficient staff training, and lower rates of patient disclosure of cannabis use (14).

A meta-analysis by Kędzior demonstrated an elevated risk of anxiety disorders among regular cannabis users, although the direction of causality appears to be partially bidirectional. Individuals with elevated neuroticism, anxiety proneness, or heightened perceptual sensitivity are particularly susceptible to derealisation reactions (24). Similar psychological vulnerability mechanisms have been observed in populations engaged in high-risk sexual behaviours and polysubstance use (45).

Evidence further indicates that anxiety sensitivity, the tendency to interpret physiological arousal as threatening, may substantially increase the likelihood of severe anxiety reactions following cannabis use. Individuals with elevated anxiety sensitivity more frequently experience anxiety, derealisation, dysphoria, or perceived loss of control, even at moderate levels of exposure. Among military veterans, regular cannabis use has been associated with higher anxiety sensitivity, poorer daily functioning, and greater severity of anxiety symptoms, suggesting that anxiety sensitivity represents a key vulnerability mechanism for adverse cannabis-related outcomes in high-risk populations (54).

More recent analyses indicate that anxiety sensitivity functions as a transdiagnostic mechanism underlying vulnerability to both panic attacks and heightened anxiety responses to psychoactive substances, including cannabis. Stewart and colleagues emphasise that, in predisposed individuals, cannabis may precipitate markedly more intense anxiety symptoms, particularly in contexts of stress, interpersonal conflict, or rapid changes in use patterns. Anxiety sensitivity may therefore partially explain why some cannabis users experience abrupt anxiety reactions, derealisation, or subclinical panic symptoms, while others report neutral or relaxing effects (55).

#### 3.1.3 Affective disorders

The association between cannabis use and depressive disorders is more complex than that observed for psychotic disorders. Nevertheless, long-term longitudinal studies indicate a moderate but clinically significant increase in the risk of depression among individuals who initiate cannabis use during adolescence (29, 37, 38). A subset of users experiences affective instability, irritability, anhedonia, and reduced motivation. These features are consistent with dysregulation of endocannabinoid and dopaminergic signalling pathways (22). High-potency cannabis products may further exacerbate mood dysregulation, producing rapid transitions between transient relaxation and abrupt dysphoria (16).

Several cohort studies, particularly from the United Kingdom, France, and Nordic countries, have reported an increased risk of suicidal ideation and suicide attempts among young cannabis users. These findings are consistent with the results of a recent meta-analysis focusing on adolescents aged 11–21 years, which demonstrated a significantly elevated risk of both suicidal ideation and suicide attempts among young cannabis users (56).

A meta-analysis of longitudinal studies demonstrated that regular cannabis use is associated with an increased risk of developing depressive disorders, especially among younger individuals and those using high-potency products. This analysis included multiple cohorts from Europe and North America, and the association remained significant after adjustment for confounders such as baseline depressive symptoms and socioeconomic status (57). These findings suggest that exposure to THC may contribute to the initiation of depressive symptoms or the exacerbation of pre-existing affective disorders.

Another meta-analysis of observational studies indicates that depression plays a significant mediating role in the relationship between cannabis use and suicidal behaviours. Among young adults, frequent cannabis use was associated with both a higher incidence of depressive episodes and an increased propensity for suicidal ideation and attempts. The analysis included populations from multiple countries, predominantly in North America and Western Europe, making its conclusions relevant to the European context, where similar risk patterns are observed. The authors emphasise that depression may function both as a moderating and mediating factor in the effects of THC, underscoring the need for particular caution in populations with increased vulnerability to affective disorders (58). At the same time, data from Southern and Central and Eastern Europe remain limited due to weaker monitoring systems, although available clinical descriptions suggest comparable patterns (4, 33).

Evidence also indicates a clinically relevant association between cannabis use and the course of bipolar disorder. Individuals with bipolar disorder who use cannabis experience more frequent symptom relapses, poorer mood stabilisation, a more severe course of depressive episodes, and greater functional impairment. A meta-analysis focusing on patients with bipolar disorder demonstrated that cannabis use is associated with a significantly increased risk of depressive episodes, greater severity of manic symptoms, less favourable treatment outcomes, and a higher likelihood of rehospitalisation (59). These findings suggest that cannabis may act as a destabilising factor in the course of bipolar disorder, which is of particular clinical importance given the increasing availability of high-potency THC products in Europe.

#### 3.1.4 Cognitive disorders and attentional dysfunction

Cannabis use exerts measurable and clinically relevant effects on cognitive functioning, particularly on working memory, attention, learning, and processing speed (23, 60). The most pronounced cognitive deficits are consistently observed among daily users, individuals who initiate use during adolescence, and those exposed to high-potency cannabis products, reflecting both dose-dependent and neurodevelopmental vulnerability effects (61). Longitudinal cohort studies indicate that early and sustained exposure to Δ9-THC interferes with normal maturation of prefrontal and hippocampal circuits, which are critical for executive control, memory consolidation, and attentional regulation (27, 60).

Neuroimaging studies conducted predominantly in Western Europe have demonstrated structural and functional alterations in the hippocampus and prefrontal cortex, including reduced grey matter volume, altered cortical thickness, and disrupted functional connectivity within fronto-hippocampal and fronto-striatal networks (26, 27, 62). Functional MRI studies further suggest impaired recruitment of executive control networks during tasks requiring sustained attention, inhibitory control, and cognitive flexibility among heavy cannabis users, particularly those using high-THC products (23). Although partial cognitive recovery has been observed after several weeks of abstinence, evidence indicates that prolonged and intensive exposure, especially to high-potency THC, may result in persistent neuropsychological impairments affecting working memory, attentional capacity, and processing speed (60, 63).

In Central and Eastern European countries, including Poland, available research data are consistent with these findings, despite more limited epidemiological coverage. Neuropsychological studies indicate that heavy cannabis users exhibit reduced working memory capacity, impaired selective attention, slower information processing speed, and diminished inhibitory control, with deficits being more pronounced among individuals using high-potency products (14, 34). The most substantial impairments are observed in tasks requiring sustained working memory, rapid attentional switching, and cognitive flexibility, functions that are essential for academic performance, occupational functioning, and everyday decision-making (14). Importantly, these cognitive disturbances may persist even in the absence of acute intoxication, suggesting long-term neurofunctional consequences rather than transient effects.

Emerging evidence further indicates that cognitive impairments associated with cannabis use frequently coexist with anxiety disorders, affective disorders, and substance-induced psychotic disorders, complicating clinical presentation and contributing to underrecognition in routine psychiatric practice (4, 25). From a public health perspective, the growing availability of high-potency cannabis products in Europe raises concerns that cannabis-associated cognitive disorders may increasingly contribute to educational underachievement, reduced work productivity, and long-term functional disability, particularly among young adults.

#### 3.1.5 Cannabis withdrawal syndrome and cannabis use disorder

Cannabis withdrawal syndrome is a formally recognized diagnosis in both Diagnostic and Statistical Manual of Mental Disorders, Fifth Edition (DSM-5) and ICD-11 and is characterized by irritability, insomnia, dysphoria, anxiety, decreased appetite, and intense craving for the substance (20, 30).

CUD is estimated to affect approximately 1–3% of adult Europeans, but as many as 30–40% of individuals who use cannabis daily (4, 32, 64). Data from the Global Burden of Disease (GBD) study confirm these observations. In Europe, particularly in Western and Northern regions, the disability burden associated with cannabis use disorder, measured as years lived with disability (YLD), is among the highest worldwide and is concentrated primarily among young adults aged 15–39 years. In Central and Eastern Europe, the estimated burden is lower; however, this is partly attributable to underdetection of cases and limited monitoring systems rather than true differences in prevalence (65).

High-potency cannabis products are associated with more severe withdrawal symptoms, greater loss of control over use, and an increased risk of relapse (19). Western European countries, including France, Spain, the Netherlands, and Germany, report the highest number of treatment entries related to cannabis use disorder (5, 35). In contrast, substantially fewer cases are registered in Central and Eastern Europe, largely due to limited availability of addiction treatment services, stigma, lack of systematic screening, and low clinical awareness (14, 33). The increasing potency of THC in the region suggests that the burden of CUD is likely to rise more rapidly in the future than indicated by official statistics.

Underdiagnosis of cannabis use disorder represents one of the major clinical challenges in Europe, particularly in its Central and Eastern regions. In many countries in this region, addiction treatment services rarely employ standardized diagnostic tools or systematic assessment of psychiatric comorbidity. In practice, this leads to delayed diagnoses, insufficient referral to psychiatric care, and omission of psychotic, anxiety, and depressive symptoms associated with cannabis use, despite its steadily increasing prevalence (7). In Poland, an additional challenge is the insufficient clinical preparation of physicians to work with patients presenting cannabis-related disorders, as demonstrated in survey-based studies conducted among medical professionals (66). Experts emphasize the need to develop early intervention pathways and strengthen integration between primary care, psychiatry, and addiction treatment services in order to shorten time to diagnosis and improve treatment outcomes (32, 67).

The scale of underdiagnosis is particularly evident in Central and Eastern European countries, including Poland, Romania, Bulgaria, Hungary, and Lithuania. Despite increasing availability of high-potency cannabis products, facilitated by online markets and cross-border distribution, clinical systems register only a small fraction of cannabis-related cases. Stigmatization of illicit substances contributes to lower rates of disclosure by patients, while clinicians often lack training to recognize characteristic clinical presentations, including THC-induced psychosis, panic attacks associated with high-potency cannabis, withdrawal symptoms, or full-syndrome cannabis use disorder (49).

Diagnostic challenges are further compounded by inconsistencies in medical documentation. In hospital settings, cases of acute psychosis are frequently classified as primary psychotic disorders without consideration of substance involvement. Limited access to toxicological testing, inconsistent use of ICD-10 and ICD-11 specifiers, and low rates of routine screening contribute to systematic underestimation of the true psychiatric burden in the population (43). Recent Polish clinical analyses support these observations. In a study by Rusiński et al., a growing number of hospitalizations and outpatient presentations related to cannabis-induced psychiatric disorders were identified, including acute psychotic episodes and exacerbation of anxiety and depressive symptoms, further highlighting the scale of this gap in the Central and Eastern European region (68). As a result, the epidemiological picture of cannabis-related disorders in CEE (Central and Eastern Europe) countries is likely to be substantially underestimated relative to the actual scope of the phenomenon.

In contrast, Scandinavian countries, most notably Norway, maintain long-standing, comprehensive psychiatric registries that allow for precise identification of substance-induced psychoses and their contribution to the etiology of psychotic disorders (50). Access to such data enables far more reliable estimation of the true clinical burden than is currently possible in regions with limited epidemiological surveillance.

In summary, underestimation and underdiagnosis of cannabis use disorder in Central and Eastern Europe do not result solely from systemic constraints but also reflect training gaps, lack of diagnostic standardization, and incomplete integration of psychiatry and addiction treatment services. Strengthening early diagnostic pathways, expanding monitoring systems, and improving clinician competence are essential prerequisites for improving quality of care in the region.

**Table 2** summarizes the main categories of cannabis-associated psychiatric disorders identified in this review, together with their most characteristic clinical manifestations, strength of evidence and key supporting sources, providing a structured overview of the psychiatric burden linked to cannabis use.

**Table 2:** Main categories of cannabis-associated psychiatric disorders and their characteristic clinical manifestations, strength of evidences and supporting sources.

Category	Key clinical features / outcomes	Strength of evidence	Sources
Psychotic disorders and first-episode psychosis	Delusions, hallucinations, disorganization of thought, relapse risk, longer hospitalizations	Strong / consistent	Di Forti, 2019; Jongsma, 2018; Hines, 2020; Schoeler, 2016 (8, 9, 11, 49)
Anxiety disorders, panic attacks, and derealization	Panic attacks, generalized anxiety, derealization, tachycardia, emergency department visits	Moderate / mixed	Petrilli, 2022; Sharpe, 2020; Bahorik, 2017; Lowe, 2024 (19, 51–53)
Affective disorders	Depressive episodes, mood instability, suicidal ideation and attempts, destabilization of bipolar disorder course	Moderate / mixed	Gobbi, 2019; Lev-Ran et al., 2013, 2014; Fresán et al., 2022; Maviel et al., 2025 (37, 56–59)
Cognitive disorders	Deficits in working memory, attention, and executive functions, slower processing speed, structural brain changes and functional impairment in daily activities	Moderate	Meier, 2012; Broyd, 2016; Volkow, 2014; Lubman, 2015; Więckiewicz, 2024; Śliżuk, 2025 (23, 26, 27, 34, 60)
Cannabis withdrawal syndrome	Irritability, insomnia, dysphoria, craving, acute symptoms following cessation or reduction of use; contribution to functional impairment and increased years lived with disability (YLD)	Moderate / consistent	Schlienz, 2017; Hasin, 2016; Manthey, 2021, 2024; Shah, 2024 (3, 20, 32, 64)
Cannabis use disorder	Impaired control over use, tolerance, continued use despite harm, chronic course, psychiatric comorbidity, increased health care utilization and years lived with disability (YLD)	Strong	Hasin, 2016; Manthey, 2021, 2024; Hall, 2025; Shah, 2024 (4, 15, 32, 64)

### 3.2 The alarming increase in THC concentrations in Europe and its consequences

The rapid increase in THC concentrations in cannabis products available on European markets over the past two decades represents one of the most important drivers of the observed clinical and epidemiological consequences of cannabis use. Laboratory analyses and EMCDDA data indicate a sustained increase in THC concentrations. in both herbal cannabis and resin, alongside the increasing availability of concentrates with THC levels reaching 60–80% (2, 6). This phenomenon has been linked to a higher incidence of first-episode psychosis, acute anxiety disorders, derealization, more severe withdrawal syndromes, and the development of CUD (19, 20). However, some studies suggest that increases in THC concentrations do not always translate into more severe acute clinical presentations (43).

An increasing body of evidence indicates that the adverse effects of high-THC products are particularly pronounced in younger populations and among daily users. This heightened vulnerability is linked to greater disruption of dopaminergic and glutamatergic neurotransmission, as well as dysregulation of endocannabinoid stress-response systems (28, 39). European studies consistently emphasize that both product potency and frequency of use constitute the primary determinants of clinical risk (6, 11, 19).

The increase in THC potency represents one of the most consequential developments in European cannabis epidemiology. The average THC concentration in herbal cannabis has risen from approximately 6–10% to 15–25% (6), while resin products frequently exceed 30–40% THC (16). European analyses further confirm the rapid spread of extracts and concentrates with THC concentrations of 60–80%. Market studies demonstrate increasing availability of high-potency products across both traditional retail markets and encrypted online platforms, facilitating wider dissemination of highly potent extracts (18). These products are strongly associated with acute psychiatric crises and an elevated risk of CUD (19).

#### 3.2.1 Changes in THC potency and market profiles – regional differentiation

The increase in THC potency has created a clear epidemiological gradient across Europe. Western and Northern European countries are characterized by the highest availability of high-potency products and the highest rates of cannabis-associated psychiatric presentations, whereas Central and Eastern Europe, despite historically lower-potency markets, is currently experiencing a rapid rise in cannabis-related morbidity driven by the inflow of potent products via online markets and cross-border supply routes (14, 15, 33).

These developments coincide with a broader transformation of the cannabis market, including thedominance of high-potency varieties in cities: Amsterdam, Barcelona, and Berlinas well as the rapid spread of high-THC products into regions previously considered low-potency markets. Recent darknet market analyses demonstrate that high-THC cannabis and concentrates constitute the majority of online cannabis offerings, with prices and availability varying according to the drug policy frameworks of exporting countries (17). This highlights the growing role of online distribution channels, which bypass local regulatory systems and accelerate the diffusion of high-risk products.

At the same time, European monitoring reports confirm that the increasing market share of high-potency cannabis is not limited to traditional markets but is also observed in newly affected regions of Europe, driven by the expansion of concentrates, “wax”-type resins, and semi-synthetic products (2, 7, 69). These changes are occurring rapidly and often escape standard surveillance systems, further underscoring the importance of enhanced monitoring of online markets and early detection of emerging trends.

**Table 3** summarizes regional differences in cannabis use prevalence, dominant product profiles, and epidemiological trends across major European regions. Estimates of adult past-year use and lifetime use among adolescents aged 15–16 years are based on EMCDDA and European School Survey Project on Alcohol and Other Drugs (ESPAD) data. Western and Northern Europe are characterized by high availability of high-THC herbal cannabis and resin, with stable or increasing daily use, whereas Southern Europe shows rising use of potent resin products. Central and Eastern Europe, historically dominated by lower-potency herbal cannabis, demonstrate rapid convergence toward higher THC concentrations and accelerating prevalence trends. These regional differences reflect both market dynamics and heterogeneity in surveillance systems and should be interpreted in the context of variable diagnostic and reporting practices across Europe (2, 35, 70).

**Table 3:** Epidemiology of cannabis use and product characteristics across European regions.

Region	Example countries	Adult past-year use*	Lifetime use age 15–16**	Dominant products	Overall trend
Western Europe	FR, NL, BE, DE, CZ	14–23%	20–28%	High-THC herbal (15–22%), high-THC resin (25–35%)	Stable high or rising
Northern Europe	DK, FI	12–17%	20–25%	High-THC herbal (15–22%), emerging concentrates (40–70%)	Rising daily use
Southern Europe	ES, IT, PT	10–20%	18–27%	Potent resin (20–35%) and mid-to-high THC herbal (12–20%)	Rising, especially resin
Central Europe	PL, HU, LT	7–18%	13–20%	Herbal THC increasing (14–20%)	Accelerating upward
Eastern Europe	RO, BG	6–10%	10–17%	Historically lower potency herbal (6–12%), rapidly increasing	Early convergence

EMCDDA (2023); ESPAD Group (2020); EUDA (2025) (2, 35, 70).

FR (France), NL (Netherlands), BE (Belgium), DE (Germany), CZ (Czech Republic), DK (Denmark), FI (Finland), ES (Spain), IT (Italy), PT (Portugal), PL (Poland), HU (Hungary), LT (Lithuania), RO (Romania), BG (Bulgaria).

#### 3.2.2 Associations between THC potency, frequency of use and the risk of psychiatric disorders

Epidemiological and clinical evidence consistently demonstrates that the risk of psychiatric disorders increases with rising THC potency. Population-based studies confirm that daily use of high-potency cannabis (>15–20% THC) is associated with a four- to five-fold increased risk of first-episode psychosis, particularly in Western European cities where such products dominate the market (8, 11).

The underlying neurobiological mechanisms include, among others:

-pronounced dysregulation of dopaminergic neurotransmission,
-greater destabilization of glutamatergic signaling,
-impaired endocannabinoid modulation, promoting heightened stress sensitivity and perceptual disturbances (39, 40).

These effects are particularly pronounced among adolescents and young adults, whose central nervous system is characterized by high neuroplasticity and increased vulnerability to psychoactive substances (27). Clinical reviews further indicate that cannabidiol (CBD) may partially attenuate some of the adverse effects of THC, including anxiety, derealization, and psychotic-like symptoms; however, these effects appear to be dose-dependent and influenced by the THC-to-CBD ratio and the evidence on this interaction remains mixed and still debated in the literature (71).

Psychiatric risk is not determined solely by THC potency but is also strongly modulated by frequency of use, as observed in other substance-related disorders, heavier patterns of consumption, particularly daily or near-daily use, are associated with a steep increase in clinical risk (30, 32).

In Western European countries, especially the United Kingdom, the Netherlands, Belgium, and France, the most common and clinically relevant pattern involves the interaction of high potency combined with daily use. This interaction translates into a predominance of first-episode psychosis, acute panic episodes, and presentations of CUD (41, 42).

Historically, Central and Eastern Europe was characterized by lower-potency products and less frequent daily use. However, in recent years, parallel to increasing availability of potent cannabis strains and intensified importation, clinicians have increasingly reported more severe psychotic episodes and acute anxiety reactions among young adults (15, 34).

This phenomenon aligns with broader patterns of psychoactive substance use in Europe, including rising substance use within high-risk populations such as chemsex settings, defined as the use of psychoactive substances in a sexual context, most commonly among men who have sex with men, where high doses and frequent consumption of psychoactive substances amplify the risk of anxiety symptoms, derealization, and cognitive deterioration (45). These regional patterns are summarized in **Table 4**.

**Table 4:** Regional variation in high-potency cannabis availability and psychiatric burden in Europe (conceptual regional model).

European region	Availability of high-THC products	Quality of clinical monitoring	Documented level of psychiatric burden
Western/Northern Europe	Very high	High	High (well documented)
Southern Europe	Moderate–high	Moderate	Moderate (partially documented)
Central and Eastern Europe	Increasing, rapidly rising	Low	Substantial, but largely underdiagnosed

#### 3.2.3 Regional gradient of psychiatric burden

Findings from the EU-GEI study confirm that risks associated with high THC potency are particularly concentrated in large European metropolitan areas. Cities like London, Amsterdam, and Paris exhibit the highest proportions of first-episode psychosis attributable to cannabis use, reaching 30–50% of all new psychosis cases (8, 9). This pattern is driven by:

- intensive informal cannabis markets,
- widespread availability of very high-potency products,
- high rates of daily use among young adults,
- greater clinical detection due to advanced monitoring systems.

In cities across Southern and Central and Eastern Europe, including Palermo, Florence, Bucharest, Warsaw, and Sofia, these proportions remain lower but are increasing in parallel with improved clinical registration and a rapid rise in the potency of products available on local markets (35). In contrast, rural regions across Europe continue to be characterized by greater availability of lower-potency products and lower case reporting, largely due to weaker psychiatric infrastructure and limited access to specialized services (13).

**Figure 2:**
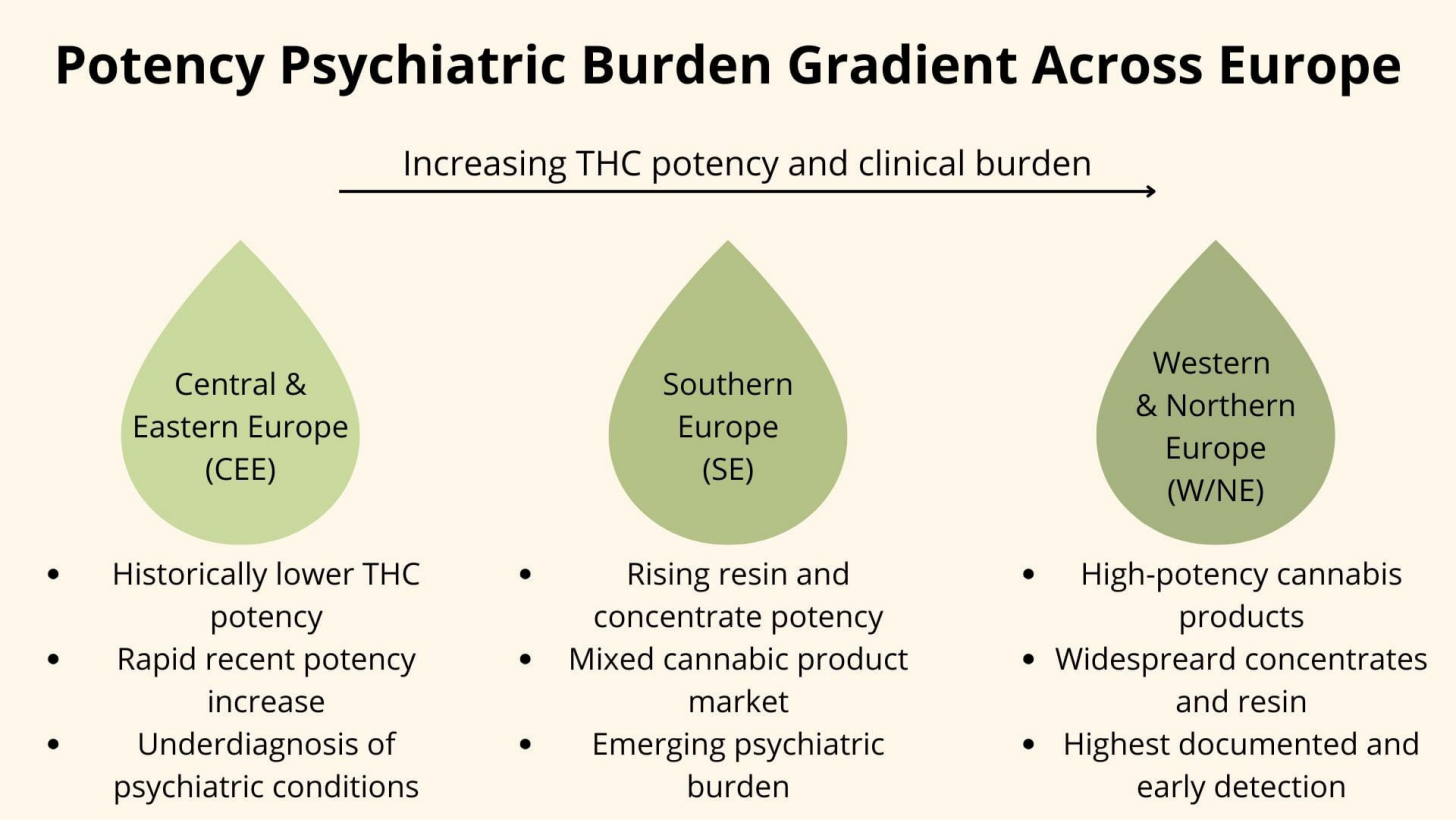
Conceptual gradient of cannabis potency, market exposure, and psychiatric burden across European regions. CEE, Central and Eastern Europe; Poland (PL); Hungary (HU); Lithuania (LT); Romania (RO); Bulgaria (BG); Czech Republic (CZ); Slovakia (SK); SE, Southern Europe; Spain (ES); Italy (IT); Portugal (PT); Greece (GR); Cyprus (CY); Malta (MT); W/NE, Western and Northern Europe; United Kingdom (UK); Netherlands (NL); France (FR); Belgium (BE); Germany (DE); Denmark (DK); Finland (FI); Sweden (SE); Norway (NO).

**Figure 2** illustrates increasing psychiatric burden associated with higher THC potency and market availability, alongside improved clinical detection in regions with more developed monitoring and mental health systems. Central and Eastern Europe is likely to experience a substantial underdiagnosed psychiatric burden due to limited surveillance capacity despite rapidly increasing THC potency. The resulting paradox is that while rising THC potency and market availability drive increasing psychiatric burden, its clinical visibility remains strongly dependent on the preparedness and diagnostic capacity of the health care system.

### 3.3 Readiness of European health care systems to respond to the rapidly changing cannabis market

The capacity of health care systems to identify, monitor, and treat cannabis-associated psychiatric disorders varies substantially across European regions. These differences reflect heterogeneity in psychiatric infrastructure, availability of addiction treatment, quality of diagnostic training, functioning of pharmacovigilance networks, and the consistency of epidemiological data collection. Western European countries generally operate more effective early-detection systems and show better integration between general psychiatry and addiction services, whereas Central and Eastern Europe (CEE) is characterized by greater system fragmentation, underdiagnosis, and limited capacity for coordinated responses (5, 35, 65). As THC potency increases and clinical presentations become more complex, these disparities are widening, revealing a growing gap between the true psychiatric burden and health system preparedness.

Overall, Western and Northern European countries (including the United Kingdom, Germany, France, Belgium, and Denmark) demonstrate the highest level of system preparedness, characterized by well-developed monitoring frameworks, comprehensive national registries, high diagnostic sensitivity, access to specialized addiction treatment services, and strong integration between psychiatry and addiction care. Southern Europe (Spain, Italy, Portugal, Greece, Malta) represents an intermediate profile, with moderate monitoring capacity, inconsistent data availability, and regional variability in access to treatment services. The lowest level of system preparedness is observed in Central and Eastern European countries (Poland, Romania, Hungary, Lithuania, Bulgaria, Latvia), which are characterized by weak surveillance systems, low detection rates, frequent misclassification of cannabis-related disorders as primary psychotic disorders, limited availability of addiction treatment services, and poor integration between psychiatry and addiction treatment services (4, 14, 33),.

#### 3.3.1 Monitoring, epidemiological surveillance, and pharmacovigilance

Western Europe maintains more advanced monitoring systems for psychoactive substance use, enabling earlier detection of cannabis-related harms. The Netherlands, Germany, France, and Portugal regularly collect data on THC potency, emergency department presentations, and cannabis-associated psychiatric disorders, integrating these indicators into national epidemiological surveillance or pharmacovigilance systems (3, 13). In addition, multicenter datasets for example, EU-GEI provide detailed information on the incidence of first-episode psychosis, allowing regional comparisons and fine-grained spatial analyses (8, 9).

Comparable standards of monitoring are observed in Scandinavia, where health systems rely on high-quality population registers. Norway operates comprehensive psychiatric and health registries that allow identification of substance-induced psychoses, including cannabis-induced psychotic disorders, and long-term trend monitoring (50). Finland employs advanced toxicological surveillance based on systematic post-mortem analyses, enabling detection of shifts in psychoactive substance exposure, including increased THC detection during periods of elevated risk (21). Sweden operates some of the most advanced toxicological and forensic programs in Europe, including Swedish STRuctured Information on Drug Abuse (STRIDA) and the Swedish National Board of Forensic Medicine systems, which generate detailed data on the presence of psychoactive substances, including cannabinoids, in clinical and forensic materials.

Switzerland represents a model of advanced product-level surveillance. Within pilot cannabis regulation programs, chemical composition, THC potency, cannabinoid profiles, and product quality are systematically analyzed in controlled retail channels. This approach complements Scandinavian clinical registries and Finnish toxicology data, forming one of the most comprehensive monitoring frameworks in Europe (72).

In contrast, Central and Eastern Europe exhibits substantial gaps in monitoring. Countries notably Poland, Romania, Bulgaria, and Hungary lack systematic registries of cannabis-associated psychiatric complications, and available data often derive from aggregated hospital statistics without substance-specific information. Stigmatization of illicit drug use, low disclosure, and limited data quality contribute to a phenomenon of “hidden prevalence,” hindering early detection of trends and increases in THC potency (14, 33). Unlike Scandinavia and Switzerland, where laboratory data on THC potency and cannabinoid exposure are routinely collected, CEE countries rely on fragmented and incomplete information (72).

#### 3.3.2 Diagnostic capacity, clinical recognition, and access to treatment

Diagnostic practices differ markedly across regions, with Western Europe, emergency departments and psychiatric units more commonly perform routine screening for cannabis use in patients presenting with acute psychosis, panic attacks, derealization, or unexplained affective instability. This facilitates earlier recognition of cannabis-induced psychotic disorder, cannabis-induced anxiety disorder, and cannabis withdrawal syndrome (43, 49).

In CEE countries, clinicians often lack specialized training in addiction psychiatry andcannabis-induced psychoses are frequently classified as primary psychotic disorders, while withdrawal symptoms remain unrecognized. This results in substantial underestimation of epidemiological burden, whilethe absence of standardized screening tools and inconsistent application of ICD-10 and ICD-11 criteria further reduce diagnostic consistency and delay therapeutic intervention (64).

Western European systems demonstrate stronger integration between general psychiatry and addiction treatment. In Germany, the Netherlands, and France, specialized outpatient and inpatient services are equipped to manage psychotic disorders alongside cannabis use disorder, supported by established referral pathways that ensure continuity of care, multidisciplinary assessment, and coordination across services (5, 35). These models align with recommendations from regulated cannabis policy analyses, which emphasize that tighter regulation facilitates access to treatment and more effective harm reduction (3).

The opposite pattern is observed in CEE, where addiction treatment remains centralized, chronically underfunded, and weakly linked to general psychiatry. There is a lack of outpatient services capable of simultaneously addressing psychotic, anxiety, and affective disorders alongside cannabis use disorder. Limited system capacity leads to delayed help-seeking, underdiagnosis, and low referral rates for CUD treatment despite increasing prevalence (14, 33).

Poland exemplifies challenges typical of the CEE region. Addiction treatment is poorly integrated with general psychiatry, and providers report substantial educational gaps regarding both pharmacotherapy and cannabis policy. Survey studies among Polish physicians demonstrate low levels of preparedness to manage patients using cannabis and underscore the need for specialized clinical pathways, highlighting systemic deficiencies in care for individuals with CUD (66).

The importance of service integration is further supported by research on social functioning. Disrupted social bonds and reduced integration, common among individuals with mental disorders, may increase vulnerability to substance-related harms and impede recovery (67). This indicates that effective CUD treatment should encompass not only pharmacological and psychotherapeutic interventions but also measures aimed at rebuilding social networks.

Despite gradual improvements in access to evidence-based interventions, many CEE countries lack unified diagnostic and therapeutic standards for cannabis-related disorders. Outpatient services are overstretched and rarely provide parallel psychiatric assessment, leading to underrecognition of CUD and delayed referral to psychiatric care, particularly in the presence of comorbid psychotic, anxiety, and affective disorders. Health policy analyses indicate that this region is characterized by limited service integration and reduced availability of preventive interventions and early-response programs (13).

Access to evidence-based treatments remains highly uneven across Europe. Cognitive-behavioral therapy, motivational interviewing, and contingency management, interventions with demonstrated efficacy for CUD, are most readily available in Western Europe, though population coverage remains variable even there (7). In Southern Europe, treatment is concentrated in large urban centers, while in CEE psychological interventions are scarce and care often consists of short-term stabilization without long-term follow-up. Services addressing withdrawal management, psychoeducation, and relapse prevention are particularly inconsistent. As high-potency products expand in CEE, the absence of structured treatment pathways promotes chronicity, functional deterioration, and increased disability burden over time (32).

Inequalities are compounded by urban–rural divides. Large metropolitan areas notably London, Amsterdam, Paris, and Berlin, identified by EU-GEI as high-incidence settings for first-episode psychosis, are supported by extensive psychiatric networks, whereas smaller cities and rural areas in Eastern and Southern Europe have limited diagnostic capacity and addiction treatment resources (8, 70).

#### 3.3.3 Mismatch between policy and diagnosis and the political gradient

Drug policies across Europe differ substantially, shaping both patterns of cannabis use and clinical outcomes. Western Europe, despite adopting more liberal approaches such as decriminalization or regulated medical access in countries like the Netherlands, Portugal, Germany, and Switzerland, simultaneously operates some of the most advanced systems of clinical and toxicological monitoring. As a result, higher recorded rates of cannabis-associated psychiatric disorders in these countries more likely reflect superior detection capacity rather than a truly higher population burden (19, 26, 72).

The literature also emphasizes that interpretation of data on cannabis use and health-related harms is often strongly dependent on the underlying drug policy model. In a re-analysis of data from 38 European countries, Stevens demonstrated that previously suggested associations between “liberalization” and increased cannabis use among adolescents were driven by flawed analytical assumptions rather than genuine epidemiological changes. The author highlights that political narratives frequently diverge from actual public health trends, further complicating risk assessment in countries with limited monitoring systems (13, 19).

Recent policy analyses indicate that Europe is entering a phase of pronounced regulatory divergence. A recent expert report by the Transnational Institute shows that EU member states are increasingly moving away from strictly prohibitionist models toward regulated forms of cannabis access, often in tension with existing UN conventions and heterogeneous EU legal frameworks (73). These differences affect not only patterns of use but also the quality of harm monitoring and the ways in which clinical cases are identified and recorded.

Consequently, countries with more liberal or regulatory approaches, often incorrectly perceived as more heavily “burdened”, may in fact possess better diagnostic, analytical, and epidemiological tools. In contrast, countries with stricter legislation, such as Poland, Romania, Hungary, or Bulgaria, report lower rates in official statistics, while limited sensitivity of surveillance systems hampers assessment of the true level of harm (15). Importantly, regional differences in cannabis-associated psychiatric morbidity reflect policy context, surveillance quality, and data availability to a degree comparable with the biological risks posed by exposure to high-THC products (4).

#### 3.3.4 Hidden threats to health care systems

Persistent data gaps in Central and Eastern Europe substantially hinder reliable estimation of the psychiatric burden associated with cannabis use. Stigmatization, restrictive legal frameworks, and chronically underfunded health information systems contribute to low reporting of cannabis involvement in cases of psychosis or anxiety episodes, a phenomenon repeatedly documented in reports on underdiagnosis of substance-related disorders in the CEE region (14, 33). These “blind spots” reduce system sensitivity and prevent timely policy responses, particularly in the context of rapidly increasing THC potency.

Western Europe, supported by more complete and detailed epidemiological data, is able to implement reforms earlier and respond more effectively to emerging clinical consequences. Higher data quality reflects more developed infrastructure, more stable funding for psychiatry, and more advanced digitalization of health care systems (5, 35). In contrast, CEE countries face workforce shortages, limited integration of electronic systems, constrained pharmacovigilance, and inconsistent reporting of psychotic disorders and cannabis use disorder, sustaining large regional disparities (15).

These inequalities mirror broader systemic differences between Western Europe and CEE in psychiatric resources, diagnostic quality, availability of addiction treatment, and implementation of public health strategies. Global data indicate that insufficient reporting markedly underestimates the burden of cannabis-related disorders, a pattern particularly evident in countries with limited epidemiological infrastructure (4). Without coordinated European standards, regional disparities are likely to deepen further as cannabis potency rises and patterns of use intensify. An effective public health response will require harmonization of reporting systems, investment in clinical training, and full integration of addiction psychiatry into mainstream mental health care, especially in Central and Eastern Europe, where current structures do not reflect the true scale of the problem.

## 4 Discussion

The findings of this review indicate that Europe is undergoing a transition in the psychiatric consequences of cannabis use, driven primarily by the increasing potency of THC concentrations and by substantial disparities in health system preparedness. Cannabis use is highest in Western and Northern Europe. High-potency products are now spreading to Southern and Central and Eastern Europe, and this is likely to increase the clinical and public health burden across the continent (4, 6).

The available evidence is not fully uniform across all outcomes. While the association between high-THC cannabis use and psychotic disorders is consistently supported across studies, findings related to anxiety, affective symptoms, and cognitive outcomes remain more heterogeneous and, in some cases, inconclusive. A structured summary of the main claims and the corresponding supporting, equivocal, and conflicting evidence is provided in **Supplementary Table 2**.

Strong cannabis products are clearly linked to psychotic symptoms. This link appears repeatedly in the literature. The EU-GEI study showed that in cities where products with more than 15–20% THC are common, such as London, Amsterdam, and Paris, daily users have a much higher risk of first-episode psychosis. In these settings, the risk was almost five times higher compared with non-users (8). High THC exposure affects brain systems involved in psychosis, as studies point to changes in dopamine and glutamate signalling. These changes increase the risk of paranoia, perceptual disturbances, and psychotic breakdown (22, 39).

High-THC cannabis is also linked to other acute psychiatric problems, with anxiety crises, panic attacks, and derealisation increasingly reported, especially in emergency departments in Western Europe (26). These effects extend beyond individuals with psychotic disorders. They are also seen in patients with anxiety disorders, mood problems, and cognitive difficulties. A key conclusion of this review concerns the uneven “visibility” of cannabis-associated psychiatric harms across Europe. Western European countries benefit from more developed registries, routine toxicological testing, more frequent use of ICD specifiers, and established early intervention networks for psychosis, enabling more accurate case identification and trend monitoring. In Central and Eastern Europe, high levels of stigma, training gaps, limited access to diagnostic tools, and fragmented data systems lead to systematic underestimation of the true psychiatric burden (14, 33). As a result, a form of “dual epidemiology” emerges: one that is well documented in Western Europe and another that remains less visible yet increasingly prevalent in CEE.

Particular attention should be paid to the interaction between THC concentrations and frequency of use. Clinical data indicate that daily use of low-potency products is associated with moderate risk, whereas daily use of high-potency cannabis generates substantially higher risk for psychosis, acute anxiety disorders, cognitive deficits, and the development of cannabis use disorder (8, 49). This finding has important public health implications, as young adults represent the group with the highest prevalence of daily use, and ongoing neurobiological maturation may amplify vulnerability to the effects of THC (26).

An additional dimension of disparity relates to urban–rural contrasts Western European metropolitan areas (London, Amsterdam, Paris, Berlin) combine highly developed high-potency cannabis markets with highly sensitive systems for detecting first-episode psychosis and cannabis use disorder. Signals from Central and Eastern European cities (including Warsaw, Bucharest, Vilnius, and Sofia) suggest a similar evolution of markets and increasing numbers of psychiatric presentations; however, these trends may not yet be fully captured by existing health care systems.

At the same time, the increasingly complex landscape of psychoactive substances in Europe complicates monitoring and interpretation of toxicological data. Biomarker studies of exposure have demonstrated substantial heterogeneity in exposure profiles across user groups, highlighting the need to develop analogous tools for THC and relevant adulterants. Moreover, research addressing complex clinical presentations and imprecise diagnostic categories (e.g., in the context of chemsex) illustrates how inadequate classification frameworks may contribute to underestimation of clinical phenomena, an issue that appears directly relevant to cannabis-related disorders.

From a health system perspective, the accumulated evidence suggests that Europe is insufficiently prepared for the consequences of rising THC potency. Even in countries with high system readiness, gaps remain in standardized screening, integration of care pathways, and clinician training. Southern Europe occupies an intermediate position, whereas Central and Eastern Europe demonstrates the lowest level of preparedness, increasing the risk of a “silent epidemic” of clinically unrecognized cases.

## 5 Future perspectives

The contemporary cannabis market has created a new epidemiological context in Europe. Rising THC potency is associated with challenges related to early detection, monitoring practices, and coordination between psychiatric services and addiction treatment systems (35). Cross-national differences remain substantial, and systems operating in isolation show limited capacity to track rapidly evolving patterns of use and the dynamics of online markets.

Key priorities should include:

-systematic monitoring of THC potency, incorporating laboratory data, analysis of online markets (including hidden and darknet channels), and surveillance of local markets (6, 18);
-routine screening for cannabis use in emergency departments, mental health outpatient services, and addiction treatment settings (42, 43);
-widespread implementation of substance-related specifiers in ICD-11, to improve the quality of registration of cannabis-associated psychotic disorders, anxiety disorders, and affective disorders (30).

In Central and Eastern Europe, diagnostic and therapeutic infrastructure remains limited in relation to the increasing exposure to high-potency cannabis products (14, 33). Availability of early intervention programs for psychosis, access to evidence-based treatments for cannabis use disorder, training opportunities for clinical staff, and outpatient addiction services varies across countries and regions (5).

At present, longitudinal data on neurobiological vulnerability to THC exposure remain limited. The long-term impact of early exposure on mental health development and the interaction between product potency, frequency of use, and psychiatric risk are not fully characterised across European regions (22, 26, 27, 40).

## 6 Limitations

This review is subject to limitations primarily related to the uneven quality of epidemiological data across Europe. Countries in Central and Eastern Europe provide particularly limited information on toxicology, diagnostic practices, epidemiology, and reporting of cannabis-associated psychiatric cases (4, 14).and lower reported rates may also reflect stigma, underreporting, and insufficient integration of health information systems.

Data on THC potency are largely derived from analyses of seized samples, which may not fully reflect consumer exposure, particularly in regions with strong online markets or in countries with lower seizure rates (6, 17). Although the association between high THC potency and psychosis is well documented, causal interpretation must account for genetic, environmental, and individual neurobiological vulnerability factors (28, 39).

As this is a narrative review, there is an inherent risk of selective interpretation. The credibility of these conclusions is strengthened by the consistency of findings across multiple European cohorts, including EU-GEI, and by their convergence with neurobiological evidence (8, 49). In addition, the lack of systematic research in many parts of Southern and Central and Eastern Europe limits the ability to fully compare regions, particularly with regard to neurocognitive studies, first-episode psychosis analyses, long-term outcomes of cannabis use disorder, and systematic monitoring of potency and use patterns.

## 7 Conclusions

High-potency cannabis is linked to mental health problems in many parts of Europe, with the strongest evidence coming from Western and Northern Europe, where exposure to high-THC products is common and data collection is more developed. In Southern Europe, use and potency are increasing, but health system responses vary. In Central and Eastern Europe, psychiatric harm is likely present, but many cases are not identified or documented.

Several patterns appear repeatedly including: higher THC levels than in the past, more frequent daily use, greater exposure among young people, differences in how countries detect and record cannabis-related problems, inconsistent diagnostic practices, limited addiction services, and weak links with psychiatric care.

Across Europe, reported data do not fully reflect clinical reality, as differences in monitoring, diagnosis, and service organisation shape what is seen in official statistics. These differences help explain why cannabis-related mental health problems appear unevenly distributed between regions.
