# Supplementary material for: Mental health consequences of contemporary cannabis use in Europe: potency, patterns of use, and health system context
Source: Front Psychiatry. 2026 Jun 10;17:1778831. doi: 10.3389/fpsyt.2026.1778831 (PMC13291135; doi:10.3389/fpsyt.2026.1778831)
Supplement: Supplementary file 1 [file DataSheet1.pdf]

Supplementary Material S1. Additional studies included in the qualitative synthesis but not individually cited in the manuscript (n = 19).

1. Babson KA, Heinz AJ, Bonn-Miller MO, et al. Cannabis and sleep architecture: a systematic review and meta-analysis. Sleep Medicine Reviews. 2025;64:102164.
2. Volkow ND, Han B, Compton WM, McCance-Katz EF. Self-reported medical and nonmedical cannabis use. Ann Intern Med. 2019.
3. Hasin DS. US epidemiology of cannabis use and associated problems. Neuropsychopharmacology. 2018.
4. Bonn-Miller MO, Babson KA, Vandrey R. Using cannabis to help you sleep: heightened frequency among those with PTSD. Drug Alcohol Depend. 2014.
5. Cooper ZD, Craft RM. Sex differences in cannabis use and effects. J Cannabis Res. 2016.
6. Lucas P, Walsh Z. Medical cannabis access and substitution patterns. Drug Alcohol Rev. 2017.
7. Pacula RL, Smart R. Medical marijuana and legalization. JAMA. 2017.
8. Degenhardt L, Hall W. Adverse health effects of cannabis use. Lancet. 2009.
9. Wang GS, Le Lait MC, Deakyne SJ, et al. Pediatric marijuana exposures. JAMA Pediatr. 2016.
10. Richards JR, Smith NE, Moulin AK. Cannabis ingestion in children. Clin Toxicol. 2017.
11. Smart R, Pacula RL. Impact of cannabis legalization. J Policy Anal Manage. 2019.
12. Harrell MB, Clendennen SL, Sumbe A, et al. Cannabis vaping among youth. Curr Addict Rep. 2022.
13. Mallory L, et al. ADHD subtypes and cannabis use. Subst Use Misuse. 2013.
14. Walsh Z, Callaway R, Belle-Isle L, et al. Cannabis for therapeutic purposes. Int J Drug Policy. 2013.
15. Hindocha C, Freeman TP, Xia JX, et al. Acute effects of cannabis. Psychopharmacology. 2017.
16. Niloy N, Hediyal TA, Chandrasekaran V, et al. Cannabis and memory. Biomolecules. 2023.
17. Cuttler C, Spradlin A, Cleveland MJ, Craft RM. Cannabis and migraine. J Pain. 2020.
18. Sorkhou M, Dent EL, George TP. Cannabis and mood disorders. Front Public Health. 2024.
19. Baltes-Flückiger L, Steinauer R, Meyer M, et al. Cannabis regulation trial protocol. Front Psychiatry. 2023
