# Supplementary material for: Mental health consequences of contemporary cannabis use in Europe: potency, patterns of use, and health system context
Source: Front Psychiatry. 2026 Jun 10;17:1778831. doi: 10.3389/fpsyt.2026.1778831 (PMC13291135; doi:10.3389/fpsyt.2026.1778831)
Supplement: Supplementary file 2 [file DataSheet2.pdf]

Table S1. Main claims and supporting evidence

Claim	Supports	Equivocal	Contradicts
Cannabis use is associated with increased risk of psychotic disorders	8, 10, 11, 48	—	—
High-potency THC cannabis increases risk of psychiatric disorders (especially psychosis)	6, 11, 19	43	—43
Early initiation and young age increase vulnerability to psychiatric outcomes	26, 27, 28, 29, 46	—	—
Cannabis use is associated with anxiety, panic reactions, and derealisation	19, 51, 52, 53	24	—
Cannabis use is associated with depressive symptoms and suicidality	29, 37, 56, 57, 58	38	—
Cannabis use worsens course of bipolar disorder and affective instability	59	—	—
Cannabis use is associated with cognitive impairment (memory, attention)	23, 60, 61, 63	—	—
High-potency cannabis increases risk of CUD and withdrawal severity	19, 30, 32, 64	—	—
THC potency and frequency of use jointly determine psychiatric risk	8, 11, 30, 32	—	—
Health system preparedness influences detection of cannabis-related disorders	5, 14, 33, 35	—	—
